# Supplementary material for: Neonatal Wnt-dependent Lgr5 positive stem cells are essential for uterine gland development
Source: Nat Commun. 2019 Nov 26;10:5378. doi: 10.1038/s41467-019-13363-3 (PMC6879518; doi:10.1038/s41467-019-13363-3)
Supplement: Supplementary file 1 — Supplementary Information [file 41467_2019_13363_MOESM1_ESM.pdf]

Supplementary Information

Neonatal Wnt-Dependent Lgr5+ Stem/Progenitor Cells are Essential for Uterine Gland Development

Seishima et al.

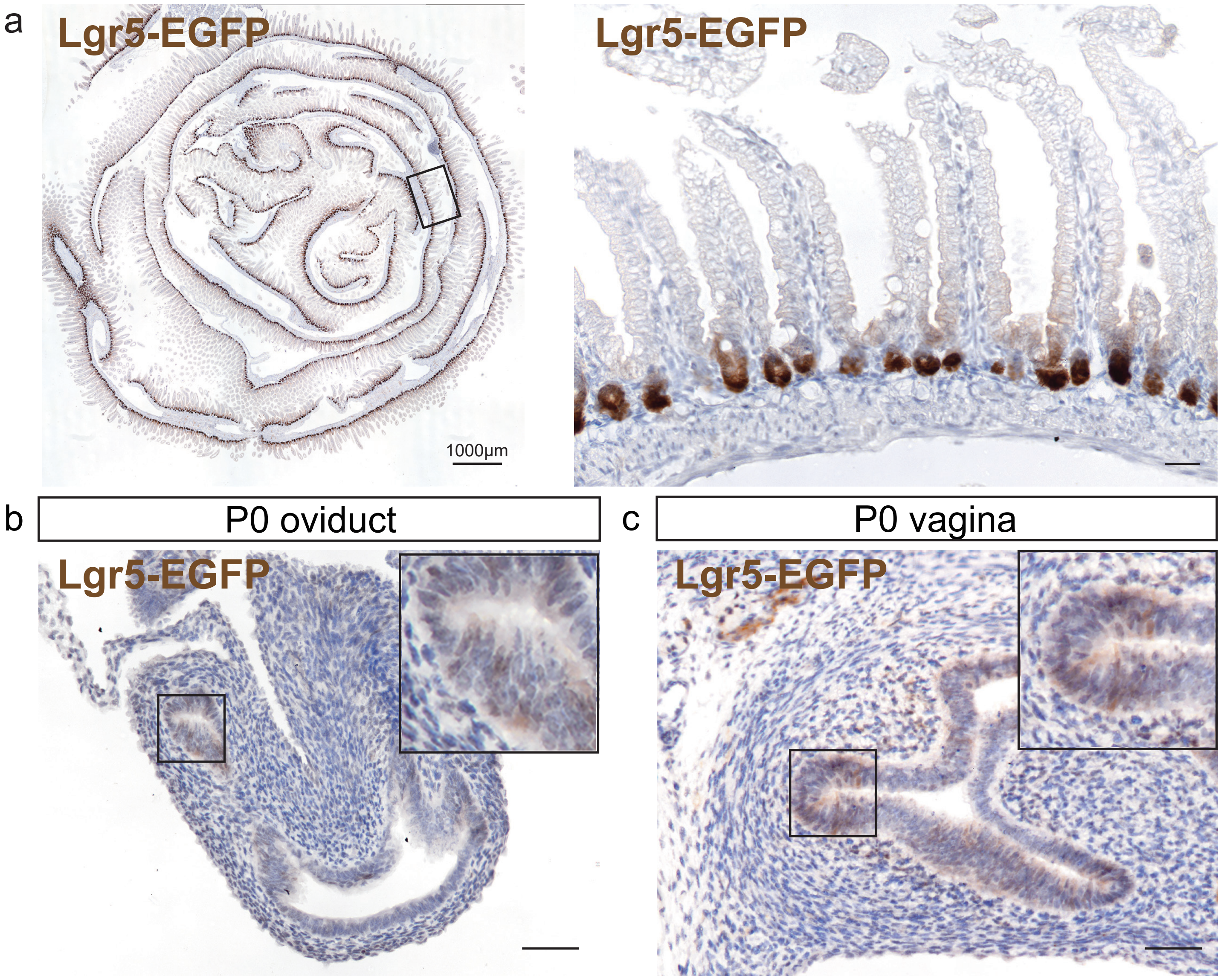

Supplementary Figure 1

a Immunostaining for Lgr5-EGFP in small intestine from P14 Lgr5-2A-EGFP mouse, shows non-variegated EGFP expression at the crypt base. **b, c** Immunostaining for Lgr5-EGFP in P0 Lgr5-2A-EGFP mouse at oviduct (b) and vagina (c). Scale bars, 50 μm. All images are representative of three independent mice.

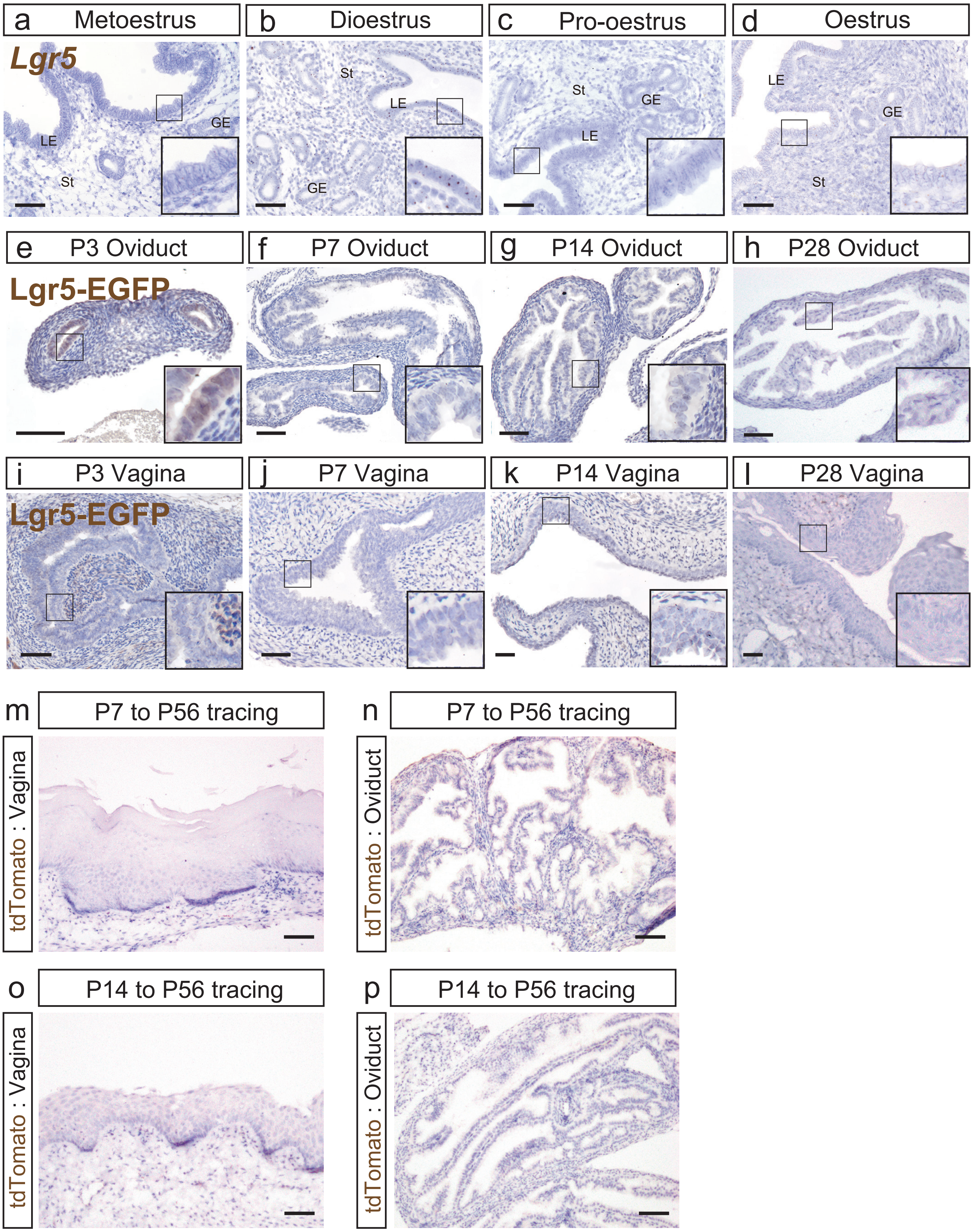

Supplementary Figure 2

a-d RNA ISH for Lgr5 in Metoestrus (a), Dioestrus (b), Pro-oestrus (c) and Oestrus (d) stage in wild-type adult uterus. LE, Luminal epithelium; GE, Glandular epithelium; St, Stroma. **e-h** IHC for Lgr5-EGFP in oviduct of Lgr5-2A-EGFP mouse at P3 (e), P7 (f), P14 (g), P28 (h). **i-l** IHC for Lgr5-EGFP in vagina of Lgr5-2A-EGFP mouse at P3 (i), P7 (j), P14 (k), P28 (l). **m-p** Immunostaining for tdTom in oviduct and vagina in Lgr5-2A-CreERT2; R26-tdTomato mouse at P56 traced from P7 (m, n) and P14 (o, p). Scale bars, 100 μm. All images are representative of three independent mice.

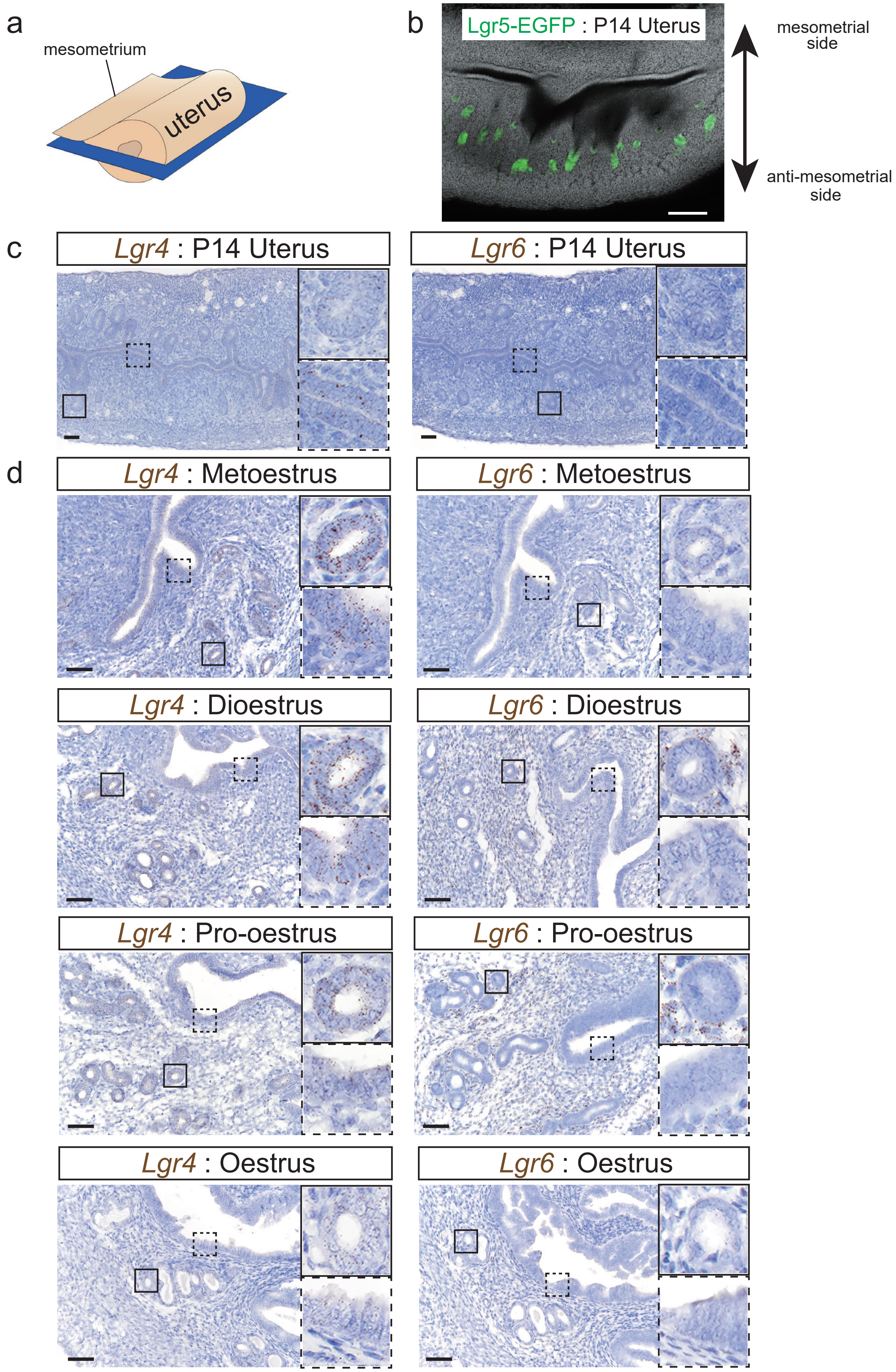

Supplementary Figure 3

a A cartoon depicting the tissue sectioning method. **b** Endogenous EGFP fluorescence in P14 Lgr5-2A-EGFP uterus. **c** RNA ISH for Lgr4 and Lgr6 on P14 wild-type uterus. **d** RNA ISH for Lgr4 and Lgr6 on wild-type adult uterus (P90) at each estrous stage. The insets with solid and dashed lines indicate GE and LE, respectively. Scale bars, 100 μm. All images are representative of three independent mice.

a

Treatment	% of mice carry to term	n-number
P7 4OHT 0.5mg/kg injected mice	100%	4
P14 TAM 150mg/kg injected mice	100%	5

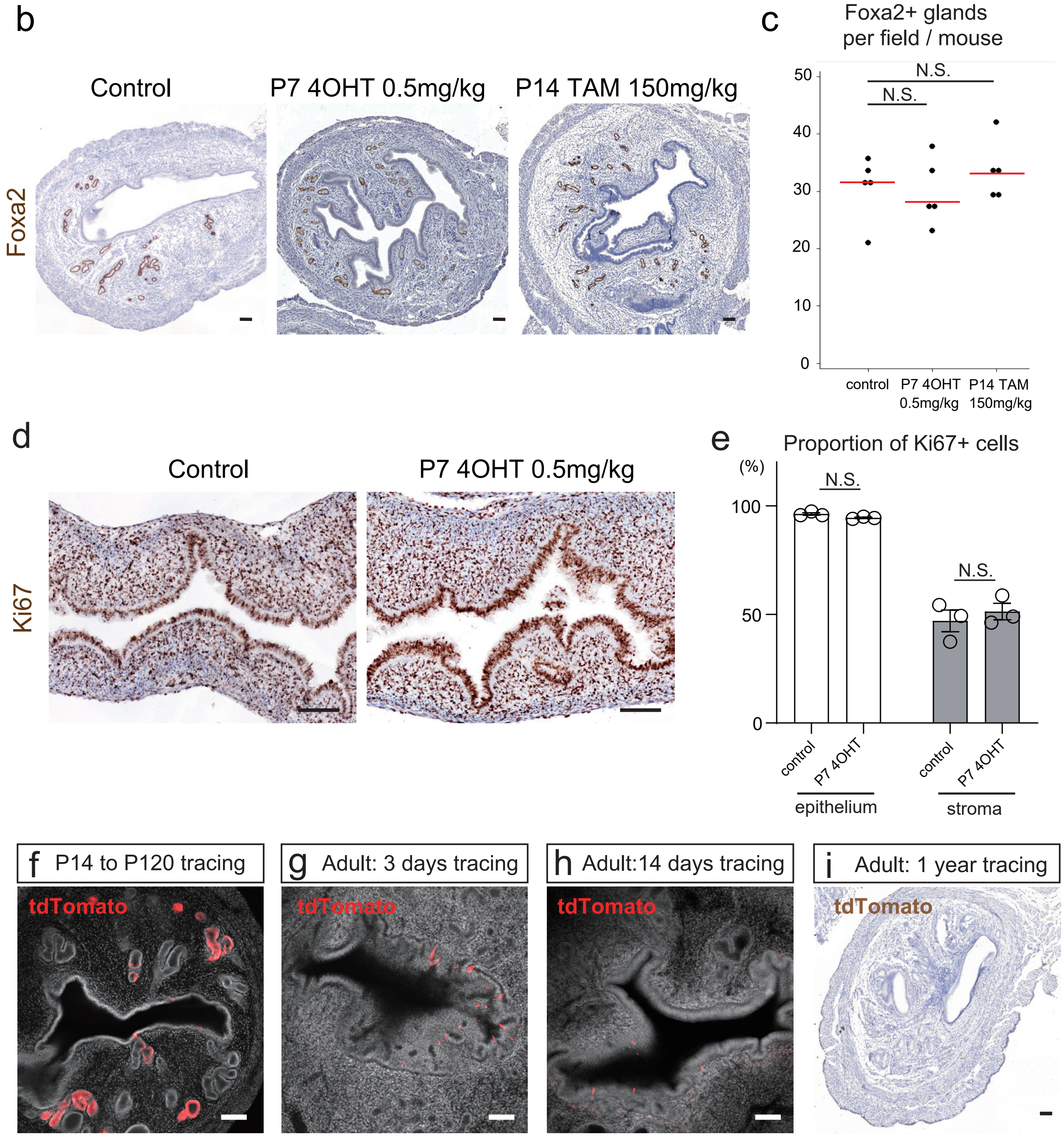

Supplementary Figure 4

a The percentages of mice carry to term from female mouse treated with 4-OHT or TAM. **b** IHC for Foxa2 in P56 WT mouse with no treatment control, treated with 4-OHT 0.5mg/kg on P7, or TAM 150mg/kg on P14. **c** Quantification of the number of Foxa2+ glands in no treatment control, 4-OHT and TAM treated mouse uterus at P56. Three independent fields from each mouse were analyzed and the average numbers of Foxa2+ glands from five independent mice are shown. Red bars represent median. Data were tested for significance using unpaired two-tailed t-test. **d** Immunostaining for Ki67 in no treatment control and 4-OHT treated P7 pups at 24h post-injection. **e** Quantitation of the percentage of Ki67+ cells in epithelium and stroma in control and 4-OHT treated P7 pups 24h after injections. Three independent fields from each mouse were analyzed and the average numbers are shown. Data from three independent experiments are presented as mean ± s.e.m. Data were tested for significance using unpaired two-tailed t-test (epithelium: $P = 0.07$, stroma: $P = 0.53$). N.S. not significant. **f** Endogenous tdTom fluorescence in Lgr5-2A-CreERT2; R26-tdTomato mouse traced from P14 to P120. **g, h** Endogenous tdTom fluorescence in Lgr5-2A-CreERT2; R26-tdTomato mouse traced for 3 (e) or 14 days (f) from adult Dioestrus stage. **i** IHC for tdTomato in Lgr5-2A-CreERT2; R26-tdTomato mouse traced for 1 year. Scale bars, 100 µm. All images are representative of three independent mice per treatment/time point.

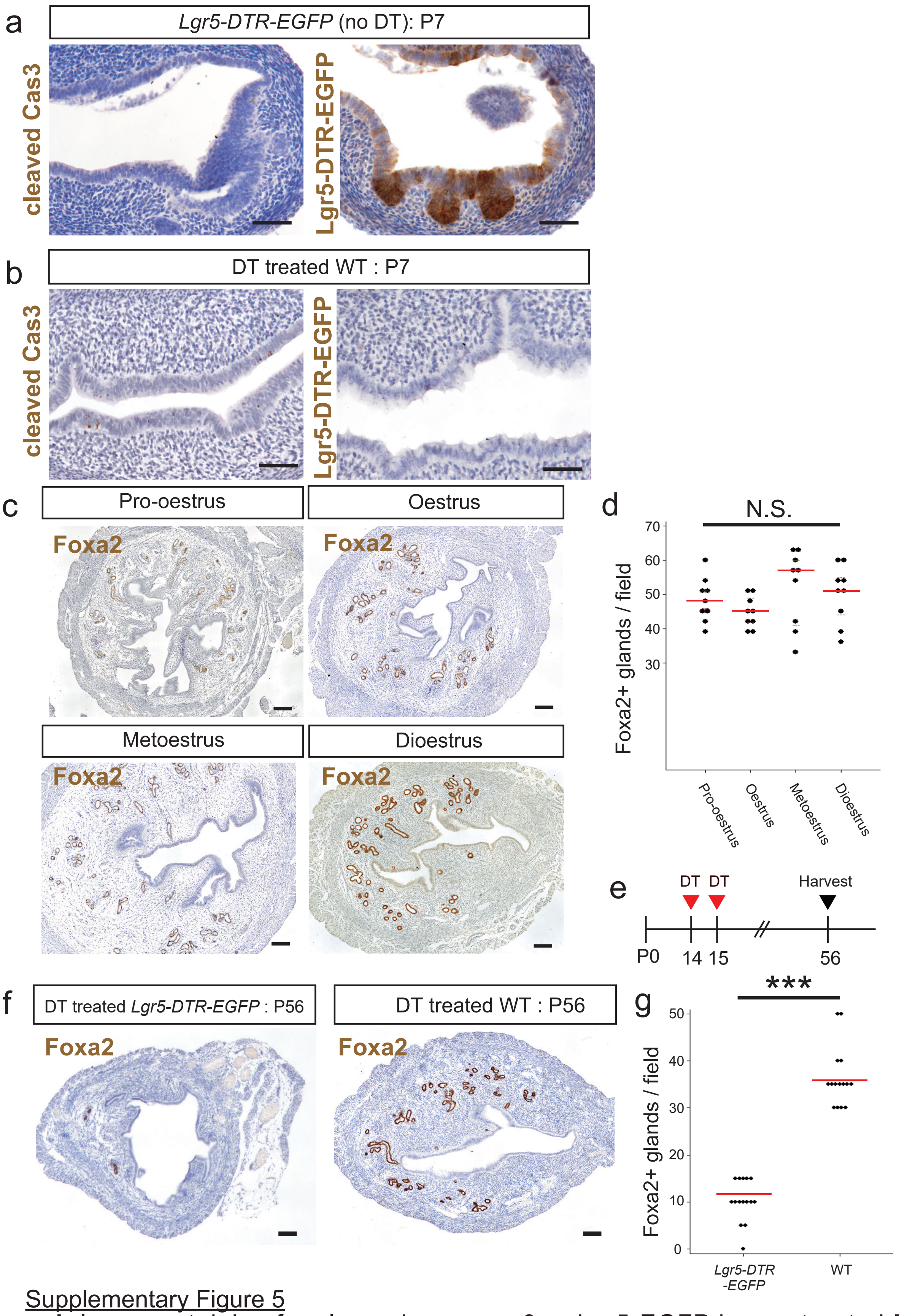

<u>Supplementary Figure 5</u>

a, b Immunostaining for cleaved caspase 3 or Lgr5-EGFP in non-treated P7 Lgr5-DTR-EGFP mouse (a) and DT treated wild-type (WT) mouse uterus (image representative of three independent mice per treatment). **c** Immunostaining for Foxa2 in adult uterus at different oestrous stages. **d** Quantification of the number of Foxa2+ glands at each oestrous stage. Three independent fields from each mouse were analyzed. Data from three independent mice are presented. Red bars represent median. Data were tested for significance by a one-way ANOVA (P = 0.32). Scale bars, 50 μm. **e** Experimental strategy for ablating Lgr5+ cells in the developing uterus. **f** IHC for Foxa2 in DT-treated Lgr5-DTR-EGFP mouse and wild type mouse (WT) at P56. Scale bars, 50 μm. **g** Quantification of the number of Foxa2+ glands in DT-treated Lgr5-DTR-EGFP and WT uterus at P56. Three independent fields from each mouse were analyzed. Data from five independent mice are presented. Red bars represent median. Data were tested for significance using unpaired two-tailed t-test (P = 3E-11). *** P < 0.001, N.S., not significant.

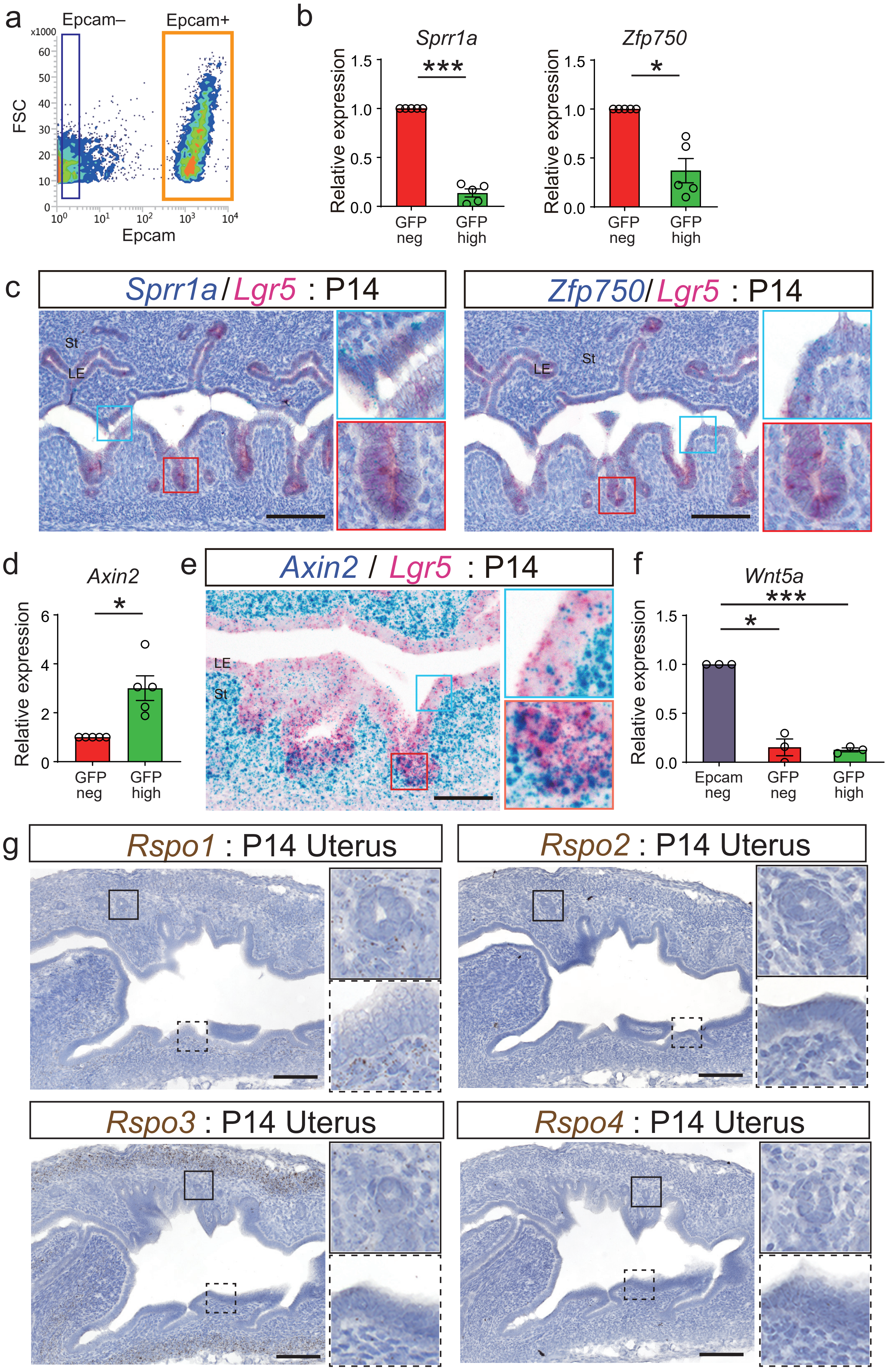

Supplementary Figure 6

a Representative FACS profile of sorted uterus epithelial cells from P14 Lgr5-2A-EGFP mouse uterus. **b** QPCR analysis of Sprr1a and Zfp750 expression on sorted EPCAM+GFPhigh cells. Data from five independent experiments are presented as mean ± s.e.m. Data were tested for significance using unpaired two-tailed t-test (Sprr1a: P = 3E-05, Zfp750: P = 7E-04). **c** RNA co-ISH of Sprr1a and Zfp750 with Lgr5 in P14 uterus. **d** QPCR analysis of Axin2 expression on sorted EPCAM+GFPhigh cells. Data from five independent experiments are presented as mean ± s.e.m. Data were tested for significance using unpaired two-tailed t-test (P = 0.016). **e** RNA co-ISH of Axin2 with Lgr5 in P14 uterus. **f** QPCR analysis of Wnt5a expression on EPCAMneg, GFPneg and GFPhigh cells sorted from P14 Lgr5-2A-EGFP mouse uterus. Data from three independent experiments are presented as mean ± s.e.m. Data were tested for significance using unpaired two-tailed t-test (Epcam neg v.s. GFP neg: P = 0.010, Epcam neg v.s. GFP high: P = 5E-04). **g** RNA ISH for Rspo1,2,3,4 on wild-type P14 uterus. The insets with solid and dashed lines indicate GE and LE, respectively. Scale bars, 200 μm. *** P < 0.001, * P <0.05

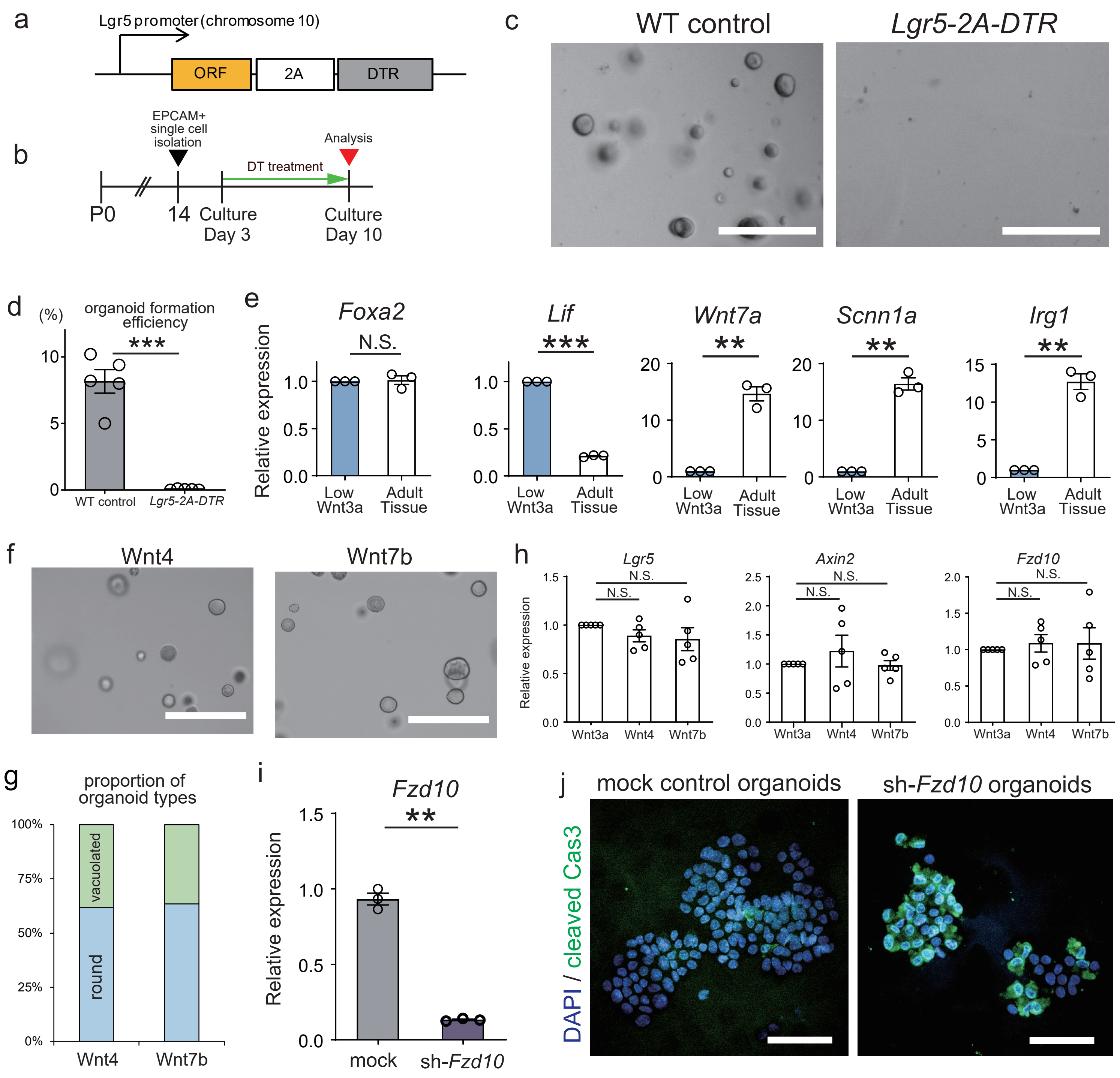

Supplementary Figure 7

a The *Lgr5-2A-DTR* mouse model employed to ablate Lgr5$^+$ cells *in vitro*. **b** Experimental strategy to ablate Lgr5$^+$ cells from *Lgr5-2A-DTR* mouse uterus *in vitro*. **c** Representative images of organoids derived from *Lgr5-2A-DTR* mouse and wild-type mouse (control) EPCAM$^+$ cells after DT treatment *in vitro* (n=5). Scale bars, 200 µm. **d** Formation efficiency of organoids derived from *Lgr5-2A-DTR* and wild-type mouse control. Data from five independent experiments are presented as mean ± s.e.m. Data were tested for significance using unpaired two-tailed t-test (P = 8E-04).**e** QPCR analysis of *Foxa2, Lif, Wnt7a*, *Scnn1a* and *Irg1* expression on sorted EPCAM$^+$GFP^{high} and EPCAM$^+$GFP^{neg} cells. Data from five independent experiments are presented as mean ± s.e.m. Data were tested for significance using unpaired two-tailed t-test (*Foxa2*: P = 0.81, *Lif*: P = 6E-05, *Wnt7a*: P = 0.008, *Scnn1a*: P = 0.005 and *Irg1*: 0.008). **f** Organoids generated from single EPCAM$^+$GFP^{high} cells cultured with Wnt4 or Wnt7b (image representative of five independent experiments). Scale bars, 200 µm. **g** The average proportions of organoid types generated under Wnt4- and Wnt7b-supplemented culture conditions from five independent experiments. **h** QPCR analysis of *Lgr5, Axin2* and *Fzd10* expression on organoids generated under each culture condition. Data from five independent experiments are presented as mean ± s.e.m. Data were tested for significance using unpaired two-tailed t-test (*Lgr5*: Wnt3a v.s. Wnt4: P = 0.15, Wnt3a v.s. Wnt7b: P = 0.29, *Axin2:* Wnt3a v.s. Wnt4: P = 0.46, Wnt3a v.s. Wnt7b: P = 0.78, *Fzd10:* Wnt3a v.s. Wnt4: P = 0.51, Wnt3a v.s. Wnt7b: P = 0.71). **i** QPCR analysis of *Fzd10* expression on mock control organoids and sh-*Fzd10* organoids. Data from three independent experiments are presented as mean ± s.e.m. Data were tested for significance using unpaired two-tailed t-test (P = 0.002). **j** Representative IF images of mock control organoids and sh-*Fzd10* organoids stained for cleaved Caspase3 (image representative of three independent experiments). Scale bars, 20 µm. *** P < 0.001, ** P < 0.01; N.S., not significant.

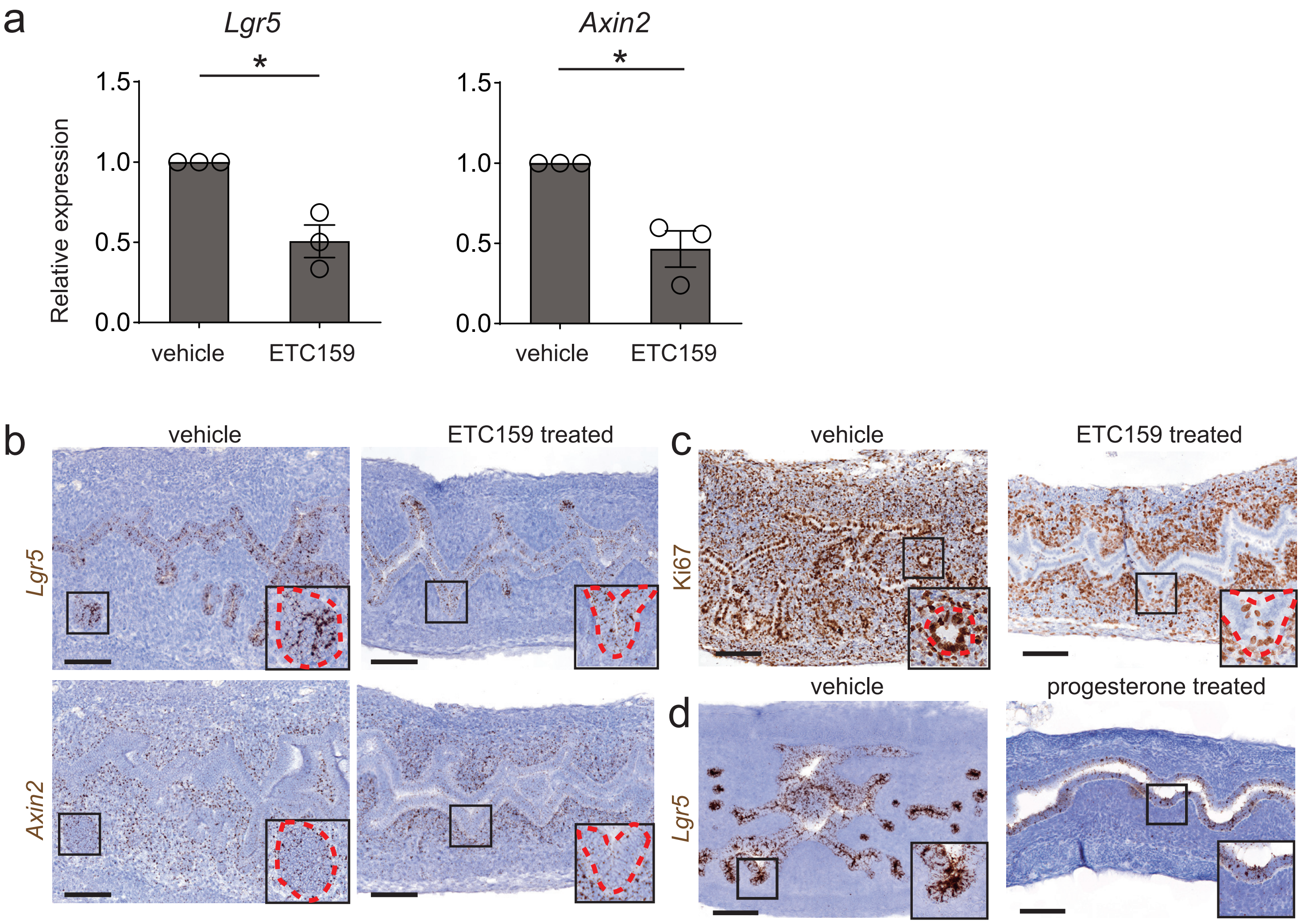

Supplementary Figure 8

a qPCR analysis of *Lgr5* and *Axin2* expression on ETC159- or vehicle-treated wild-type uterus. Data from five independent mice per treatment condition are presented as mean ± s.e.m. Data were tested for significance using unpaired two-tailed t-test (*Lgr5*: P = 0.04, *Axin2:* P = 0.04). * P < 0.05. **b** RNA ISH for *Lgr5* and *Axin2* on ETC159- or vehicle-treated wild-type uterus. **c** Immunostaining for Ki67 on ETC159- or vehicle-treated wild-type uterus. The regions encircled with dashed red lines in insets indicate developing GE (images representative of five independent mice per treatment). **d** RNA ISH for *Lgr5* on progesterone- or vehicle-treated wild-type uterus (images representative of five independent mice per treatment). Scale bars, 200 μm.

Supplementary Table 1

Differentially expressed genes in Lgr5-EGFP high versus negative cells described in Fig.6.

Gene	logFC	B	adj.P.Val
Npl	3.726369	7.23105	0.001307
Dlgap1	2.78518	6.751956	0.001613
Gstm7	2.857623	6.077227	0.003298
Plat	2.259051	5.501752	0.005836
Bche	2.211698	5.250135	0.006652
Cldn1	2.497562	5.111663	0.006704
Aldh1a1	3.522239	4.66154	0.009865
Rundc3b	2.604837	4.594059	0.009865
Sbspon	2.446463	4.402992	0.009865
Lyz2	-2.2763	4.358458	0.009865
Gas2l3	2.639892	4.298522	0.009865
Snora20	2.123699	4.28354	0.009865
Them5	1.947663	4.175369	0.010421
Lgi1	2.809467	3.86847	0.013223
Pthlh	2.387347	3.863989	0.013223
Gulo	3.162561	3.628403	0.01472
Lyrm4	1.850604	3.626538	0.01472
Rmst	1.526187	3.58529	0.01472
Tpd52l1	2.079772	3.576786	0.01472
Plek2	1.695215	3.479093	0.01507
Fra10ac1	2.069391	3.472071	0.01507
Adam28	1.81706	3.319073	0.017204
Ddt	1.557514	3.084414	0.02158
Ano2	1.832821	3.018767	0.022295
Lrpap1	1.374752	2.822601	0.025247
Prom1	2.033406	2.78418	0.025247
Zfp11	1.740713	2.767975	0.025247
Col1a1	-2.33372	2.739541	0.025247
Hscb	1.358958	2.727588	0.025247
Mia	3.615104	2.673434	0.025247
Pcp4	-3.69945	2.662636	0.025247
Scarb2	1.761915	2.654433	0.025247
Noxa1	2.243986	2.613852	0.025619
Zfp750	-1.45195	2.575422	0.025955
Ppp1r36	1.74324	2.453842	0.027825
Hook1	2.421867	2.374481	0.027825
Fkbp4	1.27761	2.371216	0.027825
Irs1	1.385761	2.352825	0.027825
Slco2a1	1.582749	2.33065	0.027825
Olfr558	-1.85533	2.30262	0.027825
Hadh	1.344818	2.254083	0.027825
Pafah1b3	1.313798	2.248095	0.027825
Mamld1	1.468829	2.234006	0.027825
Ints4	1.346317	2.211908	0.027825
Ndufa9	1.38318	2.190046	0.027825
Gpx1	1.459911	2.188979	0.027825

Gene	logFC	B	adj.P.Val
Lgr5	2.887733	2.187438	0.027825
Egfl6	1.900797	2.169185	0.027825
Arnt2	1.617653	2.158079	0.027825
Sprr1a	-4.54209	2.152703	0.027825
Ormdl1	1.52602	2.117197	0.027825
Col8a1	2.906811	2.102165	0.027825
Polr2d	2.060454	2.068528	0.027825
Cript	1.383854	2.06498	0.027825
Plet1	1.592198	2.064911	0.027825
Cdh16	1.677163	2.059247	0.027825
Nexn	-1.79898	2.017629	0.028603
Gsdmc4	-1.59343	1.991146	0.02893
Atp6v1b2	1.598777	1.942913	0.029966
Gstm2	1.432449	1.858848	0.032271
Rarres2	-3.17547	1.837703	0.032475
Upk1a	-2.0622	1.816336	0.032696
Sc5d	1.305211	1.750068	0.034555
Ddx42	1.133464	1.714577	0.034971
Lin7c	1.311324	1.709837	0.034971
4933434E20Rik	1.44639	1.692077	0.035104
Psma1	1.363509	1.639156	0.036597
2810408A11Rik	1.147747	1.621777	0.036669
Tmem29	1.177951	1.609687	0.036669
Kcne3	1.275554	1.596355	0.036669
Cd74	-1.32975	1.576244	0.036938
Ramp2	-1.21477	1.560525	0.037041
Scd1	2.753328	1.54347	0.037133
Sort1	1.485857	1.528885	0.037133
Ctsb	1.609379	1.51993	0.037133
Hdac3	1.107173	1.494521	0.03765
BC031181	1.523663	1.474142	0.037869
St3gal1	1.398272	1.464546	0.037869
Fat3	1.343719	1.435118	0.037869
Mme	2.385157	1.432511	0.037869
Mthfd1	1.280337	1.42701	0.037869
Gm8069	2.153479	1.417136	0.037869
Eef1d	1.352238	1.391361	0.037869
Mrpl17	1.200695	1.375412	0.037869
Efcab14	1.324508	1.374066	0.037869
Ciao2a	1.114066	1.372715	0.037869
Suv39h2	1.097226	1.256131	0.042291
C1qc	-1.38154	1.23922	0.042291
Aimp2	1.115338	1.195133	0.042291
Erich2	1.294783	1.185393	0.042291
Far2	2.024274	1.183565	0.042291
Pigs	1.115903	1.178674	0.042291

Gene	logFC	B	adj.P.Val
Cfh	-1.46136	1.173541	0.042291
Tmem164	1.379447	1.172603	0.042291
Trpc6	-1.5802	1.166173	0.042291
Pwp1	1.145114	1.164066	0.042291
Selenoh	1.245828	1.137874	0.042435
Snhg7	1.759156	1.13328	0.042435
Sgpp2	1.162914	1.131587	0.042435
2610524H06Rik	2.676922	1.103625	0.04274
Rpl30	1.32943	1.090877	0.04274
Akr1c19	1.885396	1.090652	0.04274
Madcam1	-1.27062	1.087097	0.04274
Lypla1	1.979779	1.05897	0.042881
Jam2	2.412918	1.036339	0.042881
Mrpl15	1.355815	1.035054	0.042881
Supt3	1.129326	1.034234	0.042881
Ccdc18	1.188568	1.029918	0.042881
Psma2	1.413065	1.023923	0.042881
Prdx2	1.016905	1.021307	0.042881
Cox7a2l	1.18115	1.001646	0.043286
2410015M20Rik	1.648267	0.993112	0.043286
Aldh1a3	1.377497	0.986656	0.043286
Rdh10	1.803289	0.96699	0.043562
Cav1	-2.67777	0.954281	0.043562
Camk2n1	-1.42973	0.929339	0.043562
Mitf	2.10164	0.927897	0.043562
Mtmr10	1.673081	0.91921	0.043562
Fbxo16	2.102298	0.918046	0.043562
Cabcoco1	1.25201	0.91544	0.043562
Eif3d	1.13572	0.885414	0.043562
Adam17	1.136443	0.878217	0.043562
Mgat4b	1.186562	0.874088	0.043562
Sox18	-1.29591	0.869289	0.043562
Hist1h2ak	1.977146	0.868328	0.043562
Trappc10	1.104732	0.861311	0.043562
Lrg1	-1.07057	0.848211	0.043562
Ap3b2	1.721829	0.847984	0.043562
Lrrn4	-1.76602	0.840165	0.043562
Slc5a11	1.511024	0.824409	0.043562
Mbnl3	1.190712	0.819572	0.043562
Chst9	1.574435	0.816581	0.043562
Chek2	1.192725	0.816196	0.043562
Ttc13	1.186073	0.814644	0.043562
Slc35a4	1.208502	0.810272	0.043562
Dcaf11	1.046747	0.800301	0.043682
Hk2	1.157408	0.793512	0.043682
Tbc1d15	1.112424	0.776007	0.043964

Gene	logFC	B	adj.P.Val
Nexmif	1.071203	0.761605	0.043964
Tnfrsf19	1.278885	0.756716	0.043964
Ckb	1.027817	0.752521	0.043964
Pmp22	-1.737	0.74371	0.043964
Ostc	1.106962	0.742478	0.043964
Haus8	1.344964	0.739448	0.043964
Casp12	-1.418	0.727524	0.044206
Sft2d2	1.805205	0.712764	0.044582
Lamtor3	1.284136	0.691944	0.044657
Sel1l3	1.430747	0.689525	0.044657
Wfdc15b	2.80371	0.689455	0.044657
Tmed6	2.4413	0.68511	0.044657
Birc2	1.267262	0.663587	0.045138
Actg2	-2.19106	0.656133	0.045138
Cavin2	-1.56231	0.655022	0.045138
Cdh2	-1.88335	0.649439	0.045138
Pgm2l1	1.502981	0.629588	0.045186
Alkbh8	1.647174	0.624994	0.045186
Zcchc2	1.464938	0.61446	0.045186
Ube2e2	0.998398	0.613501	0.045186
Hist1h2bg	1.784295	0.612167	0.045186
Vcp	1.088121	0.609151	0.045186
Nae1	1.243174	0.605545	0.045186
Vti1b	1.258458	0.58598	0.045359
Enpp1	0.980859	0.584103	0.045359
Vps28	1.629524	0.584044	0.045359
Chtf8	1.319767	0.559791	0.04623
Psma7	1.013848	0.547512	0.046296
Dgat2	2.588476	0.546325	0.046296
Vdac3	1.123616	0.538283	0.046296
Gm3776	1.277208	0.535269	0.046296
Apoe	-1.54958	0.515732	0.046837
Coro2a	1.522879	0.512666	0.046837
Cftr	1.879612	0.502596	0.047052
Nf2	1.491279	0.479286	0.047409
Gprc5b	1.492408	0.479214	0.047409
Ppip5k2	1.026173	0.47854	0.047409
Dpy30	1.421178	0.438213	0.049144
AW011738	-1.19072	0.429541	0.049305
Pltp	-1.62629	0.418325	0.049599
Sh2d4a	1.063444	0.412432	0.049623

Supplementary Table 2
Primers used for genotyping and qPCR.

Genotyping Primers		
**Genotype**	Forward	Reverse
Sry	TGACTGGGATGCAGTAGTTC	TGTGCTAGAGAGAAACCCTG
**Lgr5-2A EGFP** (mutant)	AGC CTA TGA CTT GCC TTC CA	GAA CTT CAG GGT CAG CTT GC
(wildtype)	AGC CTA TGA CTT GCC TTC CA	TGA CTT GAT GGC TTT CCT TC
**Lgr5-2A-Cre** (mutant)	AGC CTA TGA CTT GCC TTC CA	CGG TTA TTC AAC TTG CAC CA
(wildtype)	AGC CTA TGA CTT GCC TTC CA	TGA CTT GAT GGC TTT CCT TC
**Lgr5-2A-DTR**	AGC CTA TGA CTT GCC TTC CA	TTC CAC ATC ATA ACC TCC TC

qPCR Primers		
**Gene**	Forward	Reverse
Gapdh	GGTGAAGGTCGGTGTGAACG	CTCGCTCCTGGAAGATGGTG
Lgr5	TCCAACCTCAGCGTCTTC	TGGGAATGTGTGTCAAAG
Axin2	CCTGACCAAACAGACGACGA	GCTTCTGCCTCGATCTCCTC
Wnt4	CGAGCACCATTACGCCTTCAAC	AGTGCATGACCTGTTCGTAGGC
Wnt5a	GGCGGCTGCGGAGACAACAT	TCGTTCCCTTTCTCTAGCGTCCAC
Wnt7a	ACAACACACACCAGTACGCCC	GTTACACTTGACGTAGCAGCACCA
Wnt7b	CCAAAACTTGCTGGACCACG	AGGTCCGTCTCCATAGGCTT
Fzd10	TGGGCAGCATGGATGTCAAT	AAAGCCGGACAGGATGAAGG
Aldh1a1	TTCCTCCTGGCGTGGTAAA	AAACACAATGCAAGGGCTC
Prom1	CTGCGATAGCATCAGACCAAGC	CTTTTGACGAGGCTCTCCAGATC
Sprr1a	CAAGGCACCTGAGCCCTGCAA	AGGCTCTGGTGCCTTAGGTTGG
Zfp750	CCGCCTTCAAACCTGTCCAAAG	AGAGTTACTGCTCCCTGTCTGG
Foxa2	GAGAACTGGAGAAGTGTGGCTG	CTGTGAGAAGGCTACGCCATAG
Lif	GGTGGAGCTGTATCGGATGG	ATTGAGCTTGACCTGGAGGC
Prss28	TGACGACATGCTTTGTGCTGGC	CCCACCTGTATCCACTTGTTGC
Scnn1a	TGATGGTGGCTTCAACGTGAGG	AGTGCAGTCTCCGTAGTTGCCT
Irg1	GGTATCATTCGGAGGAGCAAGAG	ACAGTGCTGGAGGTGTTGGAAC
