# Supplementary material for: Neonatal Wnt-dependent Lgr5 positive stem cells are essential for uterine gland development
Source: Nat Commun. 2019 Nov 26;10:5378. doi: 10.1038/s41467-019-13363-3 (PMC6879518; doi:10.1038/s41467-019-13363-3)
Supplement: Supplementary file 3 — Reporting Summary [file 41467_2019_13363_MOESM3_ESM.pdf]

nature research

Corresponding author(s): Nick Barker NCOMMS-18-38451

Last updated by author(s): 2019/10/25

# Reporting Summary

Nature Research wishes to improve the reproducibility of the work that we publish. This form provides structure for consistency and transparency in reporting. For further information on Nature Research policies, see Authors & Referees and the Editorial Policy Checklist.

## Statistics

For all statistical analyses, confirm that the following items are present in the figure legend, table legend, main text, or Methods section.

| n/a | Confirmed | |
|---|---|---|
| ☐ | ☒ | The exact sample size (*n*) for each experimental group/condition, given as a discrete number and unit of measurement |
| ☐ | ☒ | A statement on whether measurements were taken from distinct samples or whether the same sample was measured repeatedly |
| ☐ | ☒ | The statistical test(s) used AND whether they are one- or two-sided<br>*Only common tests should be described solely by name; describe more complex techniques in the Methods section.* |
| ☒ | ☐ | A description of all covariates tested |
| ☒ | ☐ | A description of any assumptions or corrections, such as tests of normality and adjustment for multiple comparisons |
| ☐ | ☒ | A full description of the statistical parameters including central tendency (e.g. means) or other basic estimates (e.g. regression coefficient) AND variation (e.g. standard deviation) or associated estimates of uncertainty (e.g. confidence intervals) |
| ☐ | ☒ | For null hypothesis testing, the test statistic (e.g. *F*, *t*, *r*) with confidence intervals, effect sizes, degrees of freedom and *P* value noted<br>*Give P values as exact values whenever suitable.* |
| ☒ | ☐ | For Bayesian analysis, information on the choice of priors and Markov chain Monte Carlo settings |
| ☒ | ☐ | For hierarchical and complex designs, identification of the appropriate level for tests and full reporting of outcomes |
| ☒ | ☐ | Estimates of effect sizes (e.g. Cohen's *d*, Pearson's *r*), indicating how they were calculated |

*Our web collection on statistics for biologists contains articles on many of the points above.*

## Software and code

Policy information about availability of computer code

| | |
|---|---|
| Data collection | N/A |
| Data analysis | N/A |

For manuscripts utilizing custom algorithms or software that are central to the research but not yet described in published literature, software must be made available to editors/reviewers. We strongly encourage code deposition in a community repository (e.g. GitHub). See the Nature Research guidelines for submitting code & software for further information.

## Data

Policy information about availability of data

All manuscripts must include a data availability statement. This statement should provide the following information, where applicable:

- Accession codes, unique identifiers, or web links for publicly available datasets
- A list of figures that have associated raw data
- A description of any restrictions on data availability

Accesion code for microarray analysis is GSE137974.

# Field-specific reporting

Please select the one below that is the best fit for your research. If you are not sure, read the appropriate sections before making your selection.

☒ Life sciences ☐ Behavioural & social sciences ☐ Ecological, evolutionary & environmental sciences

For a reference copy of the document with all sections, see nature.com/documents/nr-reporting-summary-flat.pdf

# Life sciences study design

All studies must disclose on these points even when the disclosure is negative.

| | |
|---|---|
| Sample size | The sample size was equal or larger than 3 in all cases. No method was used to calculate the sample size.<br>We analyzed all data with standard statistical methods and referred to as significant has p<0.05, where we assume a normal distribution. |
| Data exclusions | Data was not excluded from experiments unless apparent failures. |
| Replication | We replicated our studies in all studies. Experiments that suffered from any human errors were not included in the manuscript. |
| Randomization | The experiments were not randomized. |
| Blinding | There was no blinded allocation during experiments and outcome assessment. |

# Reporting for specific materials, systems and methods

We require information from authors about some types of materials, experimental systems and methods used in many studies. Here, indicate whether each material, system or method listed is relevant to your study. If you are not sure if a list item applies to your research, read the appropriate section before selecting a response.

## Materials & experimental systems

| n/a | Involved in the study | |
|---|---|---|
| ☐ | ☒ | Antibodies |
| ☒ | ☐ | Eukaryotic cell lines |
| ☒ | ☐ | Palaeontology |
| ☐ | ☒ | Animals and other organisms |
| ☒ | ☐ | Human research participants |
| ☒ | ☐ | Clinical data |

## Methods

| n/a | Involved in the study | |
|---|---|---|
| ☒ | ☐ | ChIP-seq |
| ☐ | ☒ | Flow cytometry |
| ☒ | ☐ | MRI-based neuroimaging |

## Antibodies

Antibodies used

1.mouse anti-Lim1 (Developmental Studies Hybridoma Bank, 4F2)
2.rabbit anti-Foxa2 (Cell Signaling, #8186)
3.rabbit anti-K8 (Abcam, ab53280)
4.rabbit anti-vimentin (Abcam, ab92547)
5.mouse anti-E-cadherin (BD Transduction Laboratories, 610181)
6.rabbit anti-cleaved Caspase3 (Cell Signaling, #9661)
7.rabbit anti-Ki67 (Thermofisher, MA5-14520)
8.rabbit anti-LIF (Origene, TA321468)
9.mouse anti-Ki67 (BD Transduction Laboratories, 550609)
10.chicken anti-GFP (Abcam, ab13970)
11.rabbit anti-GFP (Cell Signaling, #2956S)
12.rabbit anti-RFP (Rockland, 600-401-379)
13.mouse anti-RFP (Abcam, ab125244)

Validation

All validation statements taken from suppliers' website.
1.Immunogen: Lim2/LhxV5 (rat); recombinant. Successfully used in WB, IP, IHC, and IF.
2.Antibody recognizes endogenous levels of total FOXA2 protein and successfully used in WB, IP, IHC, and IF.
3.Immunogen: Synthetic peptide within Human Cytokeratin 8 aa 300-400 (C terminal). Controls: human breast adenocarcinona, ovarian carcinoma, breast carcinoima, colon adenocarcinoma, endometrial carcinoma and thyroid carcinoma tissue; mouse liver tissue for IHC. HT-29 and HeLa cells for IF.
4.Immunogen: Synthetic peptide within Human Vimentin aa 400 to the C-terminus (C terminal) (acetyl ). Controls: Human kidney, colon, breast adenocarcinoma, cervical carcinoma and ovarian cancer tissues, mouse brain and kidney for IHC. HeLa, human adenocarcinoma, human schlemms canal endothelium and wildtype HAP1 cells for IF.
5.Immunogen: Human E-Cadherin aa. 735-883. Successfully used in WB, IF, IHC and IP.
6. Antibody recognizes endogenous levels of the large fragment (17/19 kDa) of activated caspase-3 resulting from cleavage adjacent to Asp175 and successfully used in WB, IP, IHC, and IF.
7.Immunogen:A synthetic peptide derived from the human Ki-67 protein. Successfully used in WB, IF, IHC and IP.
8.Immunogen: Synthetic peptide corresponding to a region derived from 34-47 amino acids of Human leukemia inhibitory factor. uccessfully used in ELISA and IHC.
9.Immunogen: Immunodominant epitope of the Ki-67 protein. Successfully used in IF.
10.Immunogen: Full length protein corresponding to Aequorea victoria GFP aa 1 to the C-terminus. Controls: GFP transfected 293 cell lysate; GFP transgenic mouse colon tissue; GFP transgenic mouse liver tissue; GFP transfected 293 cells.
11.Immunogen: A synthetic peptide corresponding to the amino terminus of GFP. Successfully used in WB, IF, IHC and IP.
12.Immunogen: A Red Fluorescent Protein (RFP) fusion protein corresponding to the full length amino acid sequence (234aa)

derived from the mushroom polyp coral Discosoma. Assay by immunoelectrophoresis resulted in a single precipitin arc against anti-Rabbit Serum and purified and partially purified Red Fluorescent Protein (Discosoma). No reaction was observed against Human, Mouse or Rat serum proteins.
13. Immunogen: Recombinant full length protein corresponding to RFP. Successfully used in WB, IF, IHC and IP.

## Animals and other organisms

Policy information about studies involving animals; ARRIVE guidelines recommended for reporting animal research

| | |
|---|---|
| Laboratory animals | Lgr5-2A-EGFP and Lgr5-2A-DTR mice were generated by homologous recombination in embryonic stem cells targeting the 2A-EGFP and 2A-DTR cassette respectively, to the stop codon of Lgr5. The Rosa26 tdTomato mice were purchased from Jackson Labs. Lgr5-DTR-EGFP mouse model has been originally generated by Frederick J de Sauvage (Nature. 2011 Oct 13;478(7368):255-9). |
| Wild animals | Female C57BL/6 mice were used as wild controls. Experiments were done at indicated ages (P3, P7, P14, P28). Mice at 6 to 8 weeks were used as adult mice. |
| Field-collected samples | N/A |
| Ethics oversight | All animal experiments were approved by the Institutional Animal Care and Use Committee of Singapore. |

Note that full information on the approval of the study protocol must also be provided in the manuscript.

## Flow Cytometry

### Plots

Confirm that:

☒ The axis labels state the marker and fluorochrome used (e.g. CD4-FITC).

☒ The axis scales are clearly visible. Include numbers along axes only for bottom left plot of group (a 'group' is an analysis of identical markers).

☒ All plots are contour plots with outliers or pseudocolor plots.

☒ A numerical value for number of cells or percentage (with statistics) is provided.

### Methodology

| | |
|---|---|
| Sample preparation | Uterine horns were harvested from mice and finely chopped using scalpel blades. Minced tissue was then incubated in chelation buffer (5.6mM sodium phosphate, 8mM potassium phosphate, 96.2mM sodium chloride, 1.6mM potassium chloride, 43.4mM sucrose, 54.9mM D-sorbitol, 1mM dithiothreitol) with 5mM EDTA, 2 mg/ml collagenase I (Worthington) and 1mM DTT at 37°C for 1h. Chelation buffer containing tissue was filtered through 100µm filter mesh, and centrifuged at 720g at 4°C for 3min. The pellet was resuspended in TrypLE (Life Technologies) with DNaseI (0.8U/µl)(Sigma) and incubated at 37°C for 10min with intermittent trituration for digestion into single cells. Digestion was quenched by dilution with cold HBSS buffer. The suspension was centrifuged at 720g at 4°C for 3min. The pellet was resuspended in HBSS with 5% fetal bovine serum (FBS, Hyclone) and filtered through a 40µm strainer. |
| Instrument | BD Influx Cell Sorter (BD Biosciences) |
| Software | FlowJo |
| Cell population abundance | Approximately 2-2.5 % of all single cells collected from P14 Lgr5-2A-EGFP mouse were GFP high epithelial cells. We characterized these cells as Lgr5 highly expressing cells by confirming the enrichment of Lgr5 expression in GFP high cells by qPCR. Data is shown in Figure 6d. |
| Gating strategy | Live cells were first extracted by gating propidium iodide negative (80%). Then the epithelial cells were extracted by gating Epcam high (30% of live cells. The plot shown in Supplementary Fig.6a). The GFP high (15%) and negative (30%) cells were then extracted due to the GFP expression intensity (Fig. 6a). |

☒ Tick this box to confirm that a figure exemplifying the gating strategy is provided in the Supplementary Information.
